# Dual-Loaded Curcumin-Metformin Niosomes: Physicochemical Characterization and *In Vitro* Activity in Colorectal and Pancreatic Cancer Cells

**DOI:** 10.34172/apb.46556

**Published:** 2026-07-15

**Authors:** Aboalhasan Al-Yaqoobi, Sina Matalqah, Zainab Lafi, Anas Abed, Sara Al-Mahamid

**Affiliations:** ^1^Department of Pharmaceutics and Pharmaceutical Technology, Faculty of Pharmacy, Al-Ahliyya Amman University, Amman 19328, Jordan; ^2^Pharmacological and Diagnostic Research Center, Faculty of Pharmacy, Al-Ahliyya Amman University, Amman 19328, Jordan; ^3^Department of Biopharmaceutics and Clinical Pharmacy, Faculty of Pharmacy, Al-Ahliyya Amman University, Amman 19328, Jordan

**Keywords:** Bioactive compounds, Cancer, Nanocarrier, Niosomes, Repurposing

## Abstract

**Purpose:**

Colorectal cancer and pancreatic cancer represent some of the most aggressive forms of cancer. Curcumin (CUR) and Metformin (MET) have distinct pharmacokinetic profiles. MET is water-soluble, whereas CUR is highly lipophilic. These differences in their kinetic behavior may limit their synchronized therapeutic activity when used in combination. This study aimed to develop and characterize dual-loaded curcumin–MET niosomes (NIO-CUR-MET) and evaluate their physicochemical properties, drug release behavior, and in vitro anticancer, apoptotic, and anti-inflammatory effects against HCT-116 (colorectal) and PANC-1 (pancreatic) cancer cell lines.

**Methods:**

Niosomes were prepared using the ethanol injection method and characterized using dynamic light scattering. Drug loading efficiency was determined by HPLC. Drug release was assessed over 72 hours using a dialysis method at 37°C. Anticancer activity was evaluated using the MTT assay, apoptosis via Caspase-3/7 analysis, and anti-inflammatory effects by measuring TNF-α and IL-1β levels using ELISA.

**Results:**

The particle size of 152.7±54 nm, and zeta potential of −12±1.3 mV, indicating a stable and uniform nanosystem. Encapsulation efficiencies were achieved for MET (91.5%±5%) and CUR (39.8%±7%). Release was observed ~95% for CURand ~92% for MET over 72 hours. The NIO-CUR-MET formulation significantly enhanced cytotoxicity compared to free and single-drug forms, with lower IC₅₀ values in HCT-116 (4±3.2 µM for CUR and 190±10.2 µM for MET) and PANC-1 cells IC₅₀ values (5.2±0.9 µM for CUR and 202.5±8.2 µM for MET). It also induced a 5.3-fold increase in apoptosis (*P*<0.001).

**Conclusion:**

Dual-loaded niosomes improved drug delivery performance and exhibited enhanced therapeutic efficacy against target cancer cell lines

## Introduction

 Cancer remains a critical global health burden concern and is among the topmost causes of death. Among all the types of cancer that are diagnosed, colorectal cancer is considered one of the most common and contributes greatly towards cancer-related deaths. On the other hand, pancreatic cancer is relatively less common but is highly aggressive in nature. Although significant progress has been made in traditional cancer treatment modalities like chemotherapy, radiation therapy, and surgery, their efficacy still leaves much to be desired. This can be attributed to several factors, including drug resistance, systemic toxicity, and non-selective nature of tumours.^1^

 CUR(CUR) is a natural polyphenolic compound derived from the turmeric plant (*Curcuma longa*) and belongs to the diarylheptanoid class, commonly known as diferuloylmethane. Its chemical structure features conjugated aromatic rings that impart a distinct yellow color and the ability to participate in electron transfer reactions, contributing to its antioxidant activity. However, this largely hydrophobic nature also leads to poor aqueous solubility, which significantly restricts its bioavailability after systemic administration.^2-4^

 Given its well-established polyphenolic nature, CUR has been extensively investigated for a broad range of pharmacological activities. These include antioxidant, anticarcinogenic, antimicrobial, anti-inflammatory, hypoglycemic, hepatoprotective, and neuroprotective effects.^5^ CUR is shown to have an anti-cancer impact, as it inhibits the growth and reproduction of several cancer cell lines. ^6,7^ Its mechanisms of action cover the suppression of inflammatory pathways, ^8^ lowering of apoptosis resistance. ^9^ and suppression of angiogenesis. ^10^

 Metformin has a relatively simple chemical structure and is commonly described as 1,1-dimethylbiguanide hydrochloride. Its biological activity is closely related to its structural conformation, which influences its pharmacokinetic behaviour, including its elimination. Although its structure may appear somewhat hydrophobic, MET is in fact highly hydrophilic and readily soluble in water. Over time, increasing research attention has been directed toward MET, revealing promising potential beyond its conventional use. While it is primarily known as an antidiabetic agent, MET has also demonstrated potential anticancer activity. Mechanistically, MET exerts its effects mainly through activation of AMP-activated protein kinase (AMPK), a key cellular energy sensor.^11,12^

 Especially, the studied niosomal system allows for controlling and sustained release when it comes to cancer treatment by overcoming most of the difficulties associated with traditional drug delivery techniques.^13^ Their nanoscale size allows for the application of the enhanced permeability and retention (EPR) effect, which facilitates preferential accumulation in tumor tissues with leaky blood vessels. ^14^ Niosomes, by definition, are vesicular drug delivery systems that can be applied for sustained, controlled, and targeted delivery. Liposomes are the oldest types of vesicular drug delivery systems; however, they suffer from limitations such as toxicity, high cost, and instability at various pH levels. ^15^

 A key aspect of formulating CUR and MET in niosomes is taking advantage of the anticancer of these agents in pancreatic and colorectal cancers. CUR acts on several signalling cascades by reducing inflammation, scavenging reactive oxygen species, inducing apoptosis, and inhibiting cell proliferation.^16^ Conversely, MET’s main activity involves activation of AMPK, which blocks the mTOR pathway, thereby lowering insulin levels and reducing growth-factor signalling in cancer cells.^17^ Preclinical studies have shown that co-treatment with CUR and MET is effective in augmenting tumor growth inhibition in colorectal cancer models, and new evidence supports its application in pancreatic cancer as well.^18-20^

 Monotherapy with either curcumin (CUR) or metformin (MET) poses distinct clinical limitations; specifically, CUR exhibits poor aqueous solubility and undergoes rapid metabolism (Figure 1),^7^ while MET exhibits high water solubility. ^18^ Niosomal encapsulation, as described previously, combines the benefits of both agents by improving stability, solubility, and sustained release. This strategy not only helps achieve and maintain therapeutic concentrations of CUR and MET over extended periods but also reduces the required dosage. This may lead to improved patient compliance and therapeutic effectiveness, particularly for aggressive cancers like colorectal and pancreatic cancer.^18,19^

**Figure 1:**
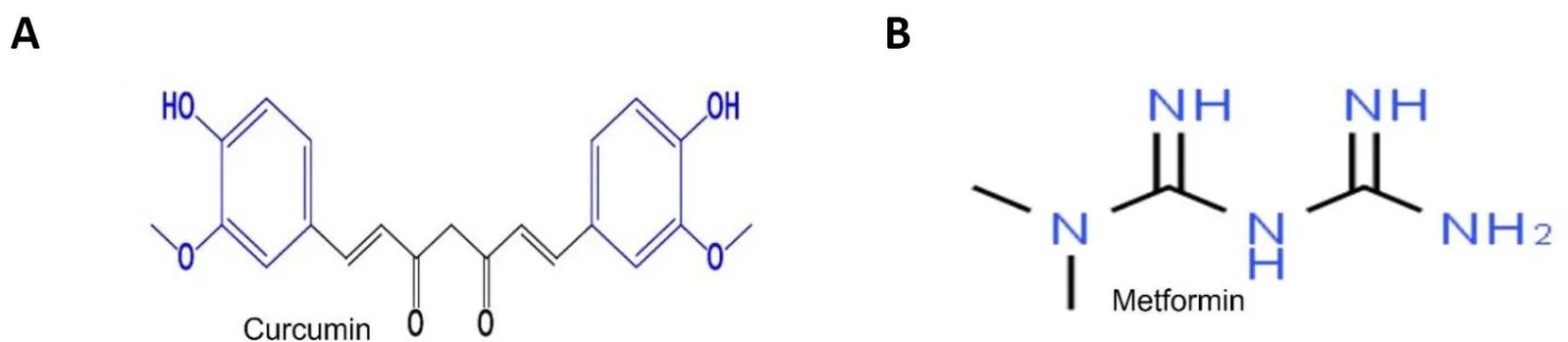
(A) Molecular structure CUR (C₂₁H₂₀O₆; molecular weight 368.38 g/mol) is a symmetric compound composed of three main structural features: two aromatic rings bearing o-methoxy phenolic groups, connected through a seven-carbon linker that contains an α, β-unsaturated β-diketone moiety. Chemical structure of MET (MET: Structure 1263). (B) Molecular structure of MET a member of the class of guanidine’s (N, N-dimethylimidodicarbonimidic diamide)

 Although, the extensive investigation of curcumin- and MET-based nanocarriers individually, the simultaneous incorporation of both agents into a single niosomal delivery system has not been previously reported. CUR and MET possess markedly different physicochemical characteristics, including differences in solubility, permeability, and bioavailability, which present substantial challenges for their synchronized delivery using conventional formulations. Therefore, the development of a co-loaded niosomal platform represents a promising strategy to overcome these limitations by enabling the simultaneous encapsulation and controlled release of both compounds within a single nanoparticle.

 In the present study, CUR and MET were co-encapsulated for the first time into niosomes with the aim of improving their stability within a single drug delivery system. This approach was designed to maximize the therapeutic potential of both agents while minimizing the limitations associated with free-drug administration. The proposed formulation was investigated as a potential therapeutic platform for colorectal and pancreatic cancers.

## Materials and Methods

 The reagents used in this research included cholesterol (> 95% purity, Sigma-Aldrich, USA), CUR (> 95% purity, Sigma-Aldrich, USA), and MET (> 98% purity, Sigma-Aldrich, USA). Absolute ethanol and methanol (HPLC grade, Fisher Scientific, USA) were used as organic solvents. The phosphate buffer solution (PBS, 1 × , pH 7.4, sterile) was obtained from Thermo Fisher Scientific (USA). Potassium bromide (KBr, FTIR grade, Sigma-Aldrich, USA) was used for IR spectrum analysis, whereas polysorbate 80 (Tween 80, analytical grade, Sigma-Aldrich, USA) was used as the surfactant for formulation development.

###  HPLC Method Development and Validation

 The high-performance liquid chromatography (HPLC) method was developed and validated for the analysis of CUR and MET.^5,21^ The analysis was performed using an HPLC system (Shimadzu, Japan) equipped with a Hypersil C18 column (4.6 × 150 mm). The detection wavelength was set at 210 nm, and the column temperature was maintained at 40°C to ensure optimal separation.

 The mobile phase was prepared from 80% methanol and 20% deionized water. The pH of the mobile phase was set to 3 by addition of 0.1 M hydrochloric acid (HCl). The flow rate was set at 1 mL/min, with an injection volume of 10 µL for each sample.

 Each sample was replicated three times, and the average of each sample was taken to plot the calibration curve. This method demonstrated reproducibility, precision, and specificity for the intended analysis.

###  Preparation of CUR–MET Niosomes (NIO-CUR-MET)

 Preparation of NIO-CUR-MET niosomes was carried out by the ethanol injection method. ^1^ Preliminary optimization formulation were performed by investigating different ratios of tween 80, cholesterol, curcumin, and MET to obtain a formulation with suitable physicochemical characteristics. The formulations were evaluated based on particle size, polydispersity index, zeta potential, encapsulation efficiency, and physical stability. The final selected composition, firstly, an aqueous phase was obtained by mixing 3 mL PBS containing MET (1 mg) and stirring the solution for about 5 minutes. Simultaneously, the lipid phase was prepared by dissolving CUR (1 mg), Tween 80 (20 μL), and cholesterol (4 mg) in 1 mL of ethanol.

 The aqueous phase was kept at 55–60°C under continuous magnetic stirring, while the lipid phase was added dropwise to it. The mixture was then stirred for 1 hour at the same temperature to facilitate the formation of niosomal vesicles.

 The resulting suspension was stored at 4°C for 24 hours prior to characterization, including measurements of particle size, zeta potential, and polydispersity index. Single-drug-loaded niosomes (NIO-MET and NIO-CUR) were prepared using the same procedure, with the only difference being the loading of either MET or CUR individually instead of the combined formulation.

###  Characterization of Prepared Niosomes

 The particle size, zeta potential, and polydispersity index (PDI) were analyzed by a Malvern Zeta Sizer. These parameters were important in determining the mean particle size (nm), surface charge (mV), and dispersion stability of the synthesized niosomal formulations. All experimental work was performed under controlled conditions in order to achieve accurate characterization of the synthesized nanomaterials.

####  Dynamic Light Scattering (DLS) and Zeta Potential Analysis

 The hydrodynamic size, zeta potential, and polydispersity index (PDI) of the prepared niosomes were analyzed by Malvern Zeta Sizer (Malvern, UK). DLS analysis was employed to determine the mean particle size (nm) and particle size distribution, while zeta potential analysis was performed to determine the mean surface charge (mV) and colloidal stability of the niosomes. In DLS analysis, 1 mL of the niosomes sample was placed in a disposable polystyrene cuvette and then analyzed at 25°C with an equilibration time of 120 seconds.

 In zeta potential analysis, 1 mL of the niosomes sample was injected into a folded capillary zeta cell without introducing air bubbles. The electrophoretic mobility of the niosomes was recorded at 25°C and zeta potential values were calculated. The resultant zeta potential values provided insight into the stability of the niosomal dispersion, where values above ± 10 mV indicated a stable colloidal system.

 PDI was also recorded as an indicator of particle size distribution, with lower values (< 0.4) suggesting a more uniform and monodisperse niosomal population.

 All experiments were performed in triplicate to ensure reliability and reproducibility. DLS analysis confirmed successful formation of niosomes and their colloidal characteristics.

####  Encapsulation Efficiency

 The encapsulation efficiency (EE%) of CUR and MET in the prepared niosomal formulations was determined by separating the unencapsulated drugs from the vesicles. To separate free CUR, the niosomal dispersion was centrifuged at 7,000 rpm for 10 min at 25°C. Concurrently, unencapsulated MET was removed via dialysis against [insert buffer name, e.g., phosphate-buffered saline] for 24 h using a dialysis membrane with a molecular weight cut-off (MWCO) of 12–14 kDa (Medicell Membranes Ltd., UK). To determine the total drug content and confirm encapsulation, the niosomes were disrupted. Briefly, a 200 μL aliquot of the niosomal formulation was mixed with 800 μL of acetonitrile to disrupt the vesicular structure, followed by probe sonication for 10 min. The mixture was then centrifuged at 10,000 rpm for 20 min, and the resulting supernatant was analyzed using the established HPLC method.

 The EE% was calculated using the following formula:


$$EE\%=\frac{Encapsulated\;drug}{Total\;Drug}*100\%$$

All formulations were prepared in triplicate under identical experimental conditions to evaluate batch-to-batch reproducibility. Following preparation, residual ethanol from the ethanol injection process was removed by continuous magnetic stirring at room temperature until complete solvent evaporation was achieved. The prepared niosomal dispersions were stored in sealed amber glass containers at 4°C and protected from light throughout the study to minimize possible degradation of CUR and MET.

####  Fourier Transform Infrared (FTIR) Spectroscopy

 FT-IR spectroscopy was employed to assess chemical interactions and verify the successful encapsulation of drugs within the niosomal formulations. The analyzed samples included CUR, MET, CUR-MET, NIO, NIO-MET, NIO-CUR, and NIO-CUR-MET.

 Approximately 1–2 mg of each solid sample was thoroughly mixed with 200 mg of potassium bromide (KBr) to obtain a uniform mixture, which was then compressed into transparent pellets under high pressure using a hydraulic press. For liquid samples (niosomal formulations), freeze-drying was performed to obtain a powder prior to mixing with KBr. FT-IR spectra were recorded using a PerkinElmer Spectrum Two spectrometer over a range of 4000–400 cm⁻¹, with a resolution of 4 cm⁻¹ and 32 scans per sample to ensure a good signal-to-noise ratio.

####  Stability Assessment of Niosomal Formulations

 A stability study was carried out to evaluate changes in the physicochemical properties of the niosomal formulations over a period of two months. After preparation and initial characterization (Section 3.5), the formulations were stored at 4°C under controlled conditions, and samples were collected at specific time intervals for analysis. The main parameters evaluated included average particle size, zeta potential, and polydispersity index (PDI), measured using dynamic light scattering (DLS) with a Malvern Zeta Sizer.

 At each time point, 1 mL of the stored niosomal suspension was equilibrated at 25°C for approximately 15 minutes before measurement. DLS analysis was then performed to determine particle size (nm) and PDI, while zeta potential (mV) was obtained from electrophoretic mobility measurements. These values were compared with initial measurements to identify any signs of instability, such as aggregation (increase in particle size or PDI) or changes in surface charge (variation in zeta potential). All measurements were conducted in triplicate to ensure reproducibility and reliability.

 This evaluation provided insight into whether the formulations maintained their original colloidal properties, supporting their suitability for extended storage and potential application.

###  In Vitro Release Studies

 An *in vitro* release study was conducted for free CUR, free MET, and the niosomal formulations (NIO-CUR-MET) using the dialysis bag diffusion method. Aliquots of the niosomal suspensions (1 mL), containing equivalent concentrations of CUR or MET, were sealed inside pre-treated dialysis bags (MWCO 12–14 kDa). For the free drug controls, (1 mg) of pure CUR or MET was dissolved or dispersed in (1 mL PBS, 7.4). To ensure complete dissolution of the highly hydrophobic free CUR and to maintain strict sink conditions for the lipophilic drug during release, (0.5% or 1% v/v) of tween 80 (50 μL/10 mL PBS) was incorporated. This non-ionic surfactant lowers interfacial tension, enhances wetting, and increases the saturation solubility of CUR in the aqueous phase, ensuring that the concentration of accumulated drug in the receiver compartment remained well below its saturation limit throughout the study.

 The free drug groups served primarily as a control to demonstrate the baseline dissolution profile and apparent solubility limitations of the free therapeutic agents under identical experimental environments. At designated time intervals (0.5, 1 2, 4, 6, 8, 24, 48, and 72 h) a 1mL aliquot of the external medium was withdrawn for analysis and immediately replenished with an equal volume of fresh, pre-warmed PBS to maintain the total volume and sink conditions. The concentration of released CUR and MET in the sampled aliquots was quantified using the established HPLC method described in Section 3.3.

 To extract the Higuchi kinetic model constants (K_H_) and linear regression coefficients (R^2^), we map the cumulative release percentage (Q_t_) against the square root of time.

 Based on the graph you provided, the release profiles exhibit a classic biphasic behavior: a rapid burst release during the first 8 hours (t = 0.5to 8h), followed by a distinct, slower controlled plateau phase from 24 to 72 hours.

 Because the Higuchi model assumes a planar matrix system where diffusion is the rate-limiting step, applying it across the entire 72-hour period will yield a poor fit due to the initial burst effect. To satisfy your reviewer, it is best practice to present the Higuchi kinetics for the sustained phase (8 to 72 h) or the initial matrix diffusion phase (0.5 to 8 h).

###  Anticancer Activity Testing (MTT)

 The human colorectal adenocarcinoma (HCT-116) and human pancreatic carcinoma (PANC-1) cell lines were evaluated in vitro anti-proliferative efficacy. Both cell lines were maintained in their respective optimal complete culture media supplemented with fetal bovine serum (FBS) and antibiotics under a humidified atmosphere of (5%CO_2_) at (37⁰ C). To initiate cultures from cryopreserved stocks, the cell vials were rapidly thawed in a water bath, immediately transferred into fresh complete medium, and centrifuged to eliminate residual cryoprotectant. The resulting cell pellets were re-suspended in fresh culture medium and transferred to standard culture flasks to allow complete metabolic recovery. Once the cultures reached (80%) confluence, subculturing was performed. Briefly, the spent medium was aspirated, the cell monolayer was washed with sterile PBS, and the cells were incubated with trypsin-EDTA to achieve complete detachment. Enzymatic activity was neutralized by adding fresh complete medium. The cell suspension was then centrifuged, and the pellet was re-suspended in fresh growth medium prior to further passaging or experimental seeding. The cytotoxicity screenings was quantified via the standard MTT assay. Cells were seeded into 96-well plates at a density of 10,000 cells per well and incubated overnight to facilitate attachment. The cells were then exposed to a gradient of concentrations (MET 100.2 μM to 3Mm while CUR 100 μM to 0.78 μM) of the free drugs and niosomal formulations alongside designated control groups (media alone, untreated, cells treated with 1% DMSO). Following the treatment 72 h, the culture medium was replaced with fresh medium containing 20 µL of a 5 mg/mL the MTT reagent stock solution, and the plates were incubated to allow viable cells to metabolize the substrate into insoluble formazan crystals. The medium was subsequently aspirated, and a solubilization solution (150 µL) was added to dissolve the intracellular formazan crystals. The absorbance of the resulting solution was quantitatively measured using a microplate reader at 570 nm wavelength. The resulting dose-response profiles of cells were used to determine the half- inhibitory concentration (IC_50_) values.

###  Apoptosis Testing

####  Protein Extraction and Quantification

 Cells HCT-116 and PANC-1 were seeded into 6-well plates (1 × 10⁶ cells/well) and allowed to attach overnight. On the next day, cells were exposed to free drug or niosomes formulation containing MET (200 μM), CUR (25 μM), and their combination for 48 h.

 Upon completion of treatments, cells were washed once with PBS, harvested with trypsin, and transferred into 15 mL-centrifuge tubes. Adherent and non-adherent cells together with the culture medium were collected. Next, the cell suspension was centrifuged at 500 × *g* for 5 min. The supernatant was removed. Cells were lysed using an adequate amount of ice-cold RIPA buffer with a protease inhibitor cocktail (Roche, UK). Cell lysates were transferred to microcentrifuge tubes and stored at -80°C until further analysis.

 The concentration of proteins was estimated using the bicinchoninic acid (BCA) assay kit (Pierce, UK). Briefly, 2 μL samples were loaded in triplicate into a 96-well plate along with a series of bovine serum albumin (BSA) standards (0.125-2 µg/mL). Afterward, 200 μL BCA working reagent (a mixture of reagents A and B in a 50:1 ratio) was added into each well. Plates were incubated at 37°C for 30 min and the absorbance was read at 570 nm using a BioTek Cytation 5 multi-mode plate reader. Protein concentrations were calculated according to the BSA standard curve.

####  Activity Assay of Caspase-3/7

 The activity of caspase-3/7 was analyzed using a colorimetric assay kit following protein extraction and quantification as described above. In brief, 50 μg of total protein per sample was added to each well of a 96-well plate. Thereafter, 50 μL of 2x reaction buffer (including 100 mM DTT) and 5 μL of DEVD-p-NA substrate (4 mM stock solution, final concentration 200 μM) were added. The mixture was gently shaken and incubated at 37°C for 1 h. The absorbance was read at 400 nm using a BioTek Cytation 5 plate reader.

###  Anti-Inflammatory Activity (TNF-α and IL-1B)

 The HCT116 and PANC-1 cells were assessed for their inflammatory response to MET and CUR exposure, either in free or encapsulated forms, using the enzyme-linked immunosorbent Assay (ELISA) technique. Cells were treated with free and niosomal formulations as follows: 40 µM of MET, 5 µM of CUR, or equivalent concentrations of the mix and incubated for a duration of 24 hours. Thereafter, 50 μl of growth media supernatant was added to the 96 well plate provided with the kit. To this, 50 μl of the antibody cocktail solution was added to each of the sample-loaded wells. The following research has several limitationsThe antibody cocktail, comprising both capture and detection antibodies, was added to the wells. The plate was then sealed to prevent any evaporation or contamination and incubated for 1 hour at room temperature. Throughout this time, the plate was consistently shaken at a speed of 400 rpm to ensure an even mixture and exposure. Once the incubation was completed, each well underwent a washing procedure three times with 350 μl of washing buffer and any residual liquid was thoroughly removed by inverting the plate and gently tapping against clean paper towels. Subsequently, 100 μl of the TMB development solution was added to each well, and the plate was incubated in a dark environment, being shaken at 400 rpm for 15 minutes. To terminate the enzymatic reaction, 100 μl of the Stop Solution was introduced to every well and the plate was shaken for an additional minute to ensure even mixing. Finally, the optical density (OD) of each sample was recorded at a wavelength of 450 nm using a spectrophotometer. The TNF-α and IL-1β concentration was interpolated in reference to a pre-established calibration curves.

###  Statistical Analysis

 All experiments were performed independently in triplicate (n = 3), and experimental data are expressed as mean standard deviation (SD). Comparisons among multiple experimental groups were analyzed using one-way ANOVA followed by Tukey’s post-hoc test for multiple comparisons. All statistical analyses were conducted using GraphPad Prism software (version 8). Statistical significance thresholds are indicated by asterisks as follows: (*P -value < 0.05), (**P -value < 0.01), (***P -value < 0.001), and (****P -value < 0.000), while (*P* -value < 0.05) was considered statistically non-significant (ns).

## Results and Discussion

###  Preparation and Characterization of Niosomes

 Niosomes were successfully fabricated using the ethanol injection method, and their physicochemical properties—hydrodynamic diameter, polydispersity index (PDI), zeta potential (ζ-potential), encapsulation efficiency (EE%), and loading efficiency (LE%)—were determined (Figure 2A–C). The hydrodynamic diameter of the vesicles ranged from 152.7 to 168.2 nm. Blank NIO measured 168.2 ± 24 nm, while drug-loaded formulations were slightly smaller: NIO-MET (158 ± 74 nm), NIO-CUR (156.2 ± 88 nm), and NIO-CUR-MET (152.7 ± 54 nm). Transition Electron microscopy (TEM) image of NIO-CUR-MET showing spherical morphology at 16,000 × magnification with a scale bar of 3 μm. (Figure 2D).

**Figure 2:**
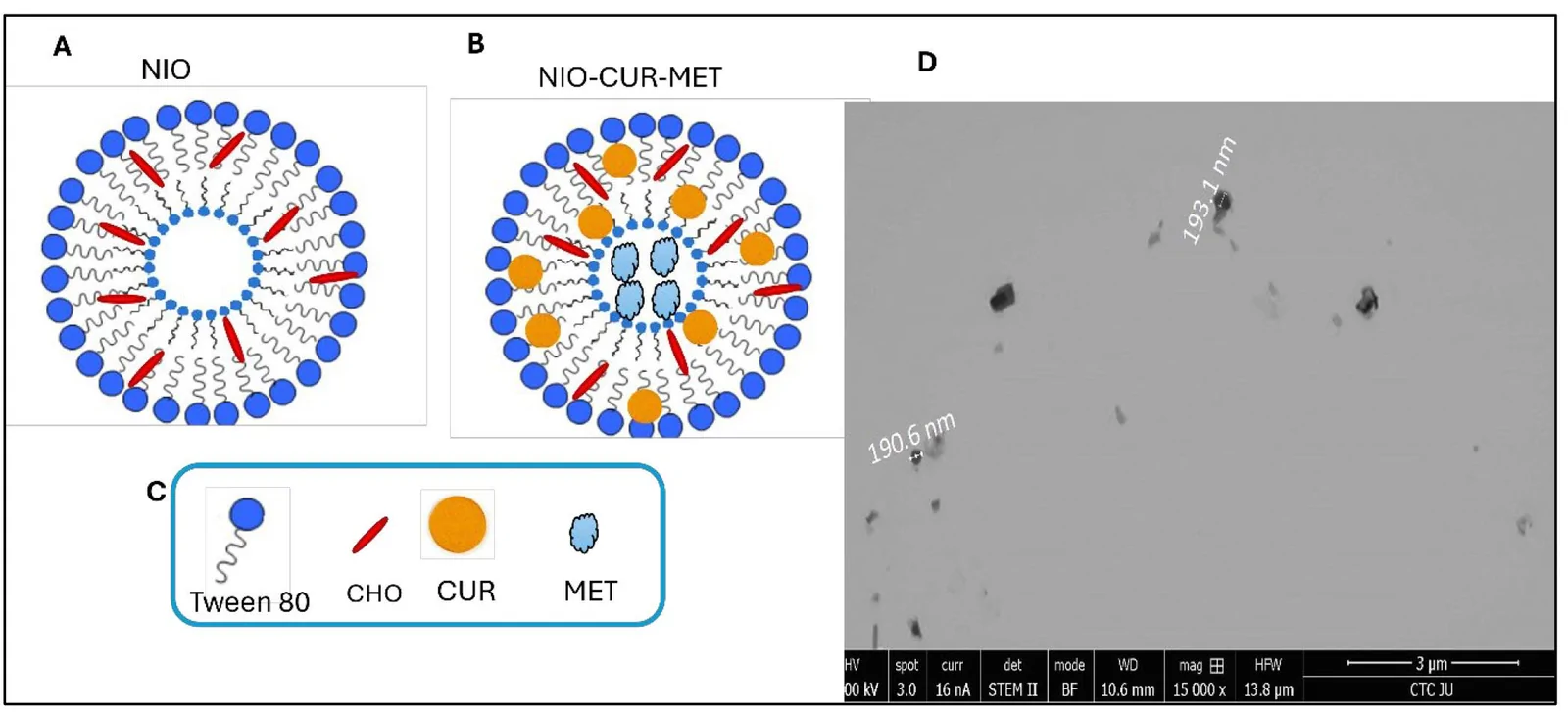
Schematic representation and morphological analysis of niosomes. (A) Structure of blank niosomes (NIO) composed of Tween 80 (blue head with gray tail) and cholesterol (CHO, red rods). (B) Structure of CUR- and MET-loaded niosomes (NIO-CUR-MET) showing CUR(CUR, orange spheres) in the bilayer and MET (MET, pink molecules) in the aqueous core. (C) Key to schematic symbols. (D) TEM image of NIO-CUR-MET showing spherical morphology at 16,000 × magnification (scale bar = 3 μm)

 The polydispersity index (PDI) values revealed distinctive variations in size uniformity among the formulations. NIO-Free exhibited the narrowest distribution (0.169 ± 0.001), followed closely by NIO-MET (0.199 ± 0.001) and NIO-CUR (0.200 ± 0.00). The dual-loaded formulation (NIO-CUR-MET) maintained a highly acceptable PDI of (0.241 ± 0.001), confirming its homogeneous nature, which is visually demonstrated by the monodisperse dynamic light scattering (DLS) size distribution peak (Figure 3D).

**Figure 3:**
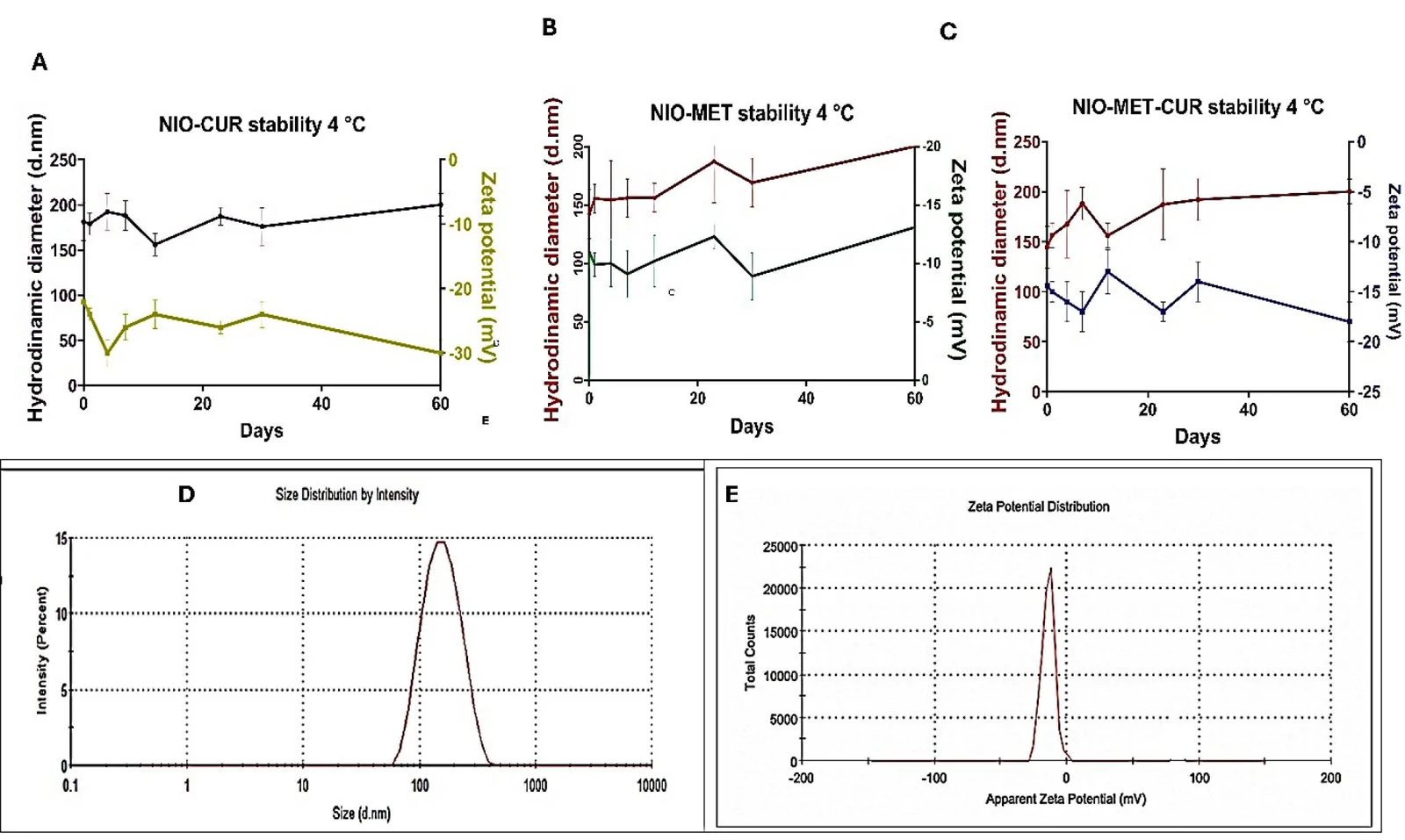
Characterization and stability of niosomes-based formulations. (A) Stability of NIO-CUR, (B) NIO-MET, and (C) NIO-MET-CUR over 60 days at 4 °C. (D) DLS particle size distribution of NIO-MET-CUR. (E) Zeta potential distribution of NIO-MET-CUR

 Surface charge analysis revealed that NIO-Free and NIO-CUR possessed the highest negative surface charges (-19 ± 4.81 mV) and (-19.3 ± 2.1 mV), respectively), reflecting strong baseline electrostatic stability. The incorporation of MET resulted in a lower absolute surface charge for both NIO-MET (-10 ± 3.58 mV) and NIO-CUR-MET (-12 1.3 mV) (Figure 3E).

 This reduction is likely due to the localization or shielding effect of the hydrophilic MET molecules at the vesicle interface. Crucially, while the lower absolute zeta potential values of the MET-containing formulations suggest weaker electrostatic repulsion compared to their counterparts, they still provide sufficient surface charge to prevent immediate aggregation. Furthermore, because these systems are composed of non-ionic surfactants, their long-term colloidal stability is heavily driven by steric hindrance provided by the outward-extended hydrophilic headgroups of the surfactants, which physically shield the vesicles from coalescing.

 This co-stabilization mechanism (electrostatic and steric forces) was firmLy validated by the stability studies conducted at 4°C over 60 days (Figure 3A–C). All formulations retained robust physical integrity over two months. NIO-CUR-MET demonstrated excellent dimensional stability, keeping a highly consistent particle size throughout the study, whereas NIO-MET and NIO-CUR experienced minor, gradual expansions slightly exceeding (200 nm) by day 60. Over the same storage period, the zeta potential profiles remained active within stabilized negative ranges, shifting from approximately (-22) to (-30 mV) for NIO-CUR, (-15) to (-18 mV) for NIO-MET, and (-10) to (-20 mV) for NIO-CUR-MET. This shift toward more negative values over time indicates a reorganization of the surfactant bilayers or drug orientation at the interface that maintained, or slightly enhanced, the electrostatic barrier. Collectively, the preserved nanoscale dimensions and stable surface charges over 60 days confirm that the prepared niosomes possess highly favorable physicochemical characteristics and structural robustness for pharmaceutical applications.

 The niosomal matrix demonstrated an exceptional capacity for dual-drug accommodation, displaying distinct preferences based on the physicochemical properties of the cargo. MET achieved a remarkably high encapsulation efficiency of (91.5% ± 5%) in the dual-loaded niosomes, significantly higher than its encapsulation in the single-loaded counterpart (49.5% ± 4%). As a highly hydrophilic small molecule, MET preferentially resides within the spacious aqueous core of the vesicles. A highly lipophilic CUR was successfully integrated into the hydrophobic domains of the surfactant bilayer, yielding an EE% of (39.8% ± 7%) in the dual-loaded matrix. This represents a substantial enhancement compared to the single-loaded CUR niosomes (17.56% ± 3%).

 The dramatic boost in encapsulation efficiency for both therapeutics during co-loading suggests a enhanced entrapment mechanism. The presence of MET within the internal aqueous core may alter the trans-membrane packing geometry or chemical potential, allowing the lipid bilayer to accommodate a higher density of hydrophobic CUR molecules without destabilizing the vesicle. Conversely, the dense packing of CUR within the hydrophobic tails may minimize water leakage from the core, sealing MET more effectively inside. This dual-delivery architecture successfully maximizes the payload for both water-soluble and water-insoluble drugs within a single vehicle (Table 1).

**Table 1:** Summary table presenting hydrodynamic diameter (nm), polydispersity index (PDI), zeta potential (mV), encapsulation efficiency (EE%), and loading efficiency (%) for NIO-Free, NIO-MET, NIO-CUR, and NIO-MET-CUR (mean ± SD, n = 3).

**Liposomes** **(n=3)**	**Hydrodynamic diameter** **(nm±SD)**	**Polydispersity index** **(PDI±SD)**	**ζ-potential** **(mV±SD)**	**EE%**	**Loading efficiency %**
NIO- Free	168.2 ± 24	0.169 ± 0.001	-19 ± 4.81	-	-
NIO-MET	158 ± 74	1.99 ± 0.001	-10 ± 3.58	49.5% ± 4%	8.3% ± 0.66%
NIO- CUR	156.2 ± 88	0.2 ± 0.001	- 19.3 ± 2.1	17.56% ± 3%	3% ± 0.4%
NIO-CUR-MET	152.7 ± 54	0.241 ± 0.001	- 12 ± 1.3	CUR: 39.8% ± 7%	CUR: 6.7% ± 1.2%
				MET: 91.5% ± 5%	MET: 15.3% ± 0.8%

 Niosomes prepared by ethanol injection method exhibited favorable physicochemical properties, with particle sizes and polydispersity index, indicating a uniform and homogenous population of vesicles. ^15^ The zeta potential values of the formulations were negative, suggesting good colloidal stability and low aggregation tendency. Importantly, the co-encapsulation of CUR and MET did not adversely affect the stability or structural integrity of the niosomes, supporting their compatibility within the same carrier system.^22^

 High encapsulation efficiency was observed, especially for CUR, likely due to its hydrophobic nature and affinity for the niosomal bilayer, while MET, being hydrophilic, showed moderate encapsulation within the aqueous core. These results are consistent with previous studies on similar systems.^23,24^ The ability of niosomes to accommodate

 both hydrophobic and hydrophilic drugs supporte their use in combination therapies and enables synchronized delivery.

 The formulations remained physically stable over 30 days at 4°C, with minimal changes in particle size. The inclusion of cholesterol in the bilayer likely contributed to the membrane rigidity and stabilization of the vesicle structure.^25^

###  HPLC Method Development

 Quantification of the analytes (CUR and MET) was performed using pre-established calibration curves. For CUR, the calibration equation was y = 5 × 10⁷x – 126,350 with R^2^ = 0.9977 and a retention time of 4.51 min. For MET, the equation was y = 3 × 10⁷x + 147,853 with R^2^ = 0.9998 and a retention time of 2.16 min (Figure 4A–C). The high correlation coefficients confirmed excellent linearity and reliability of the developed HPLC method, with negligible interference from matrix effects or instrumental variation. The lower limit of quantification (LLOQ) was 2 µg for both CUR(CUR) and MET (MET).

**Figure 4:**
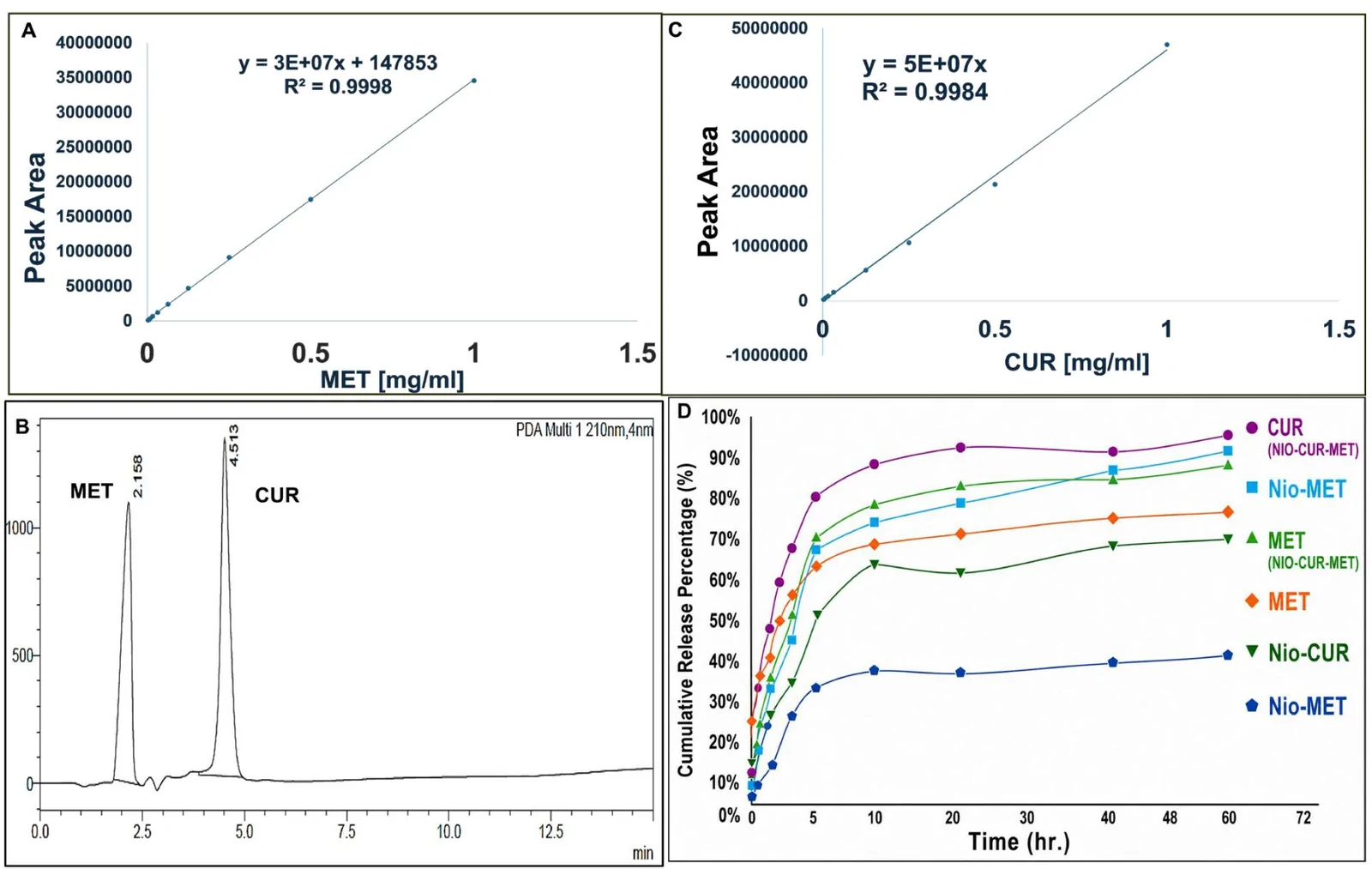
Quantitative analysis, chromatographic separation, and in vitro release profiles of MET (MET) and CUR(CUR). (A) Calibration curve of MET. (B) HPLC chromatogram of MET and CUR at 210 nm (retention times: 2.163 and 4.513 min). (C) Calibration curve of CUR. (D) In vitro release profiles of free CUR, free MET, NIO-CUR, and NIO-MET over 72 h

 The RP-HPLC method provided a reliable and effective approach for the simultaneous quantification of CUR(CUR) and MET (MET), with distinct and consistent retention times, making it suitable for evaluating dual-loaded niosomal formulations. This method is crucial for assessing drug content, encapsulation efficiency, and release kinetics in formulations containing both hydrophilic and hydrophobic drugs.^5,21^

###  In Vitro Release of CUR and MET

 The in vitro release profiles of CUR and MET, in free form, single-loaded, and dual-loaded niosomes, were evaluated over 72 hours (Figure 4D). During the initial 30 minutes, MET exhibited the highest burst release (23%), followed by NIO-MET (15%) and NIO-CUR-MET (14%), while CUR formulations showed a slower release, with NIO-CUR (12%), NIO-CUR-MET (10%), and CUR (6%). At 8 hours, CUR release from NIO-CUR-MET (83%) was markedly higher compared to NIO-CUR (61%) and CUR (35%), whereas MET release also peaked in NIO-CUR-MET (69%), slightly surpassing NIO-MET (66%) and closely matching MET-free (65%). By 24 hours, CUR release from NIO-CUR-MET reached 92%, significantly higher than NIO-CUR (59%) and CUR-free (35%), while MET release followed a similar pattern, with NIO-CUR-MET (79%) exceeding both NIO-MET (75%) and MET (69%). At the 72-hour endpoint, dual-loaded niosomes exhibited the highest cumulative release for both drugs, with CUR at 95% (vs. 71% in NIO-CUR and 39% in CUR) and MET at 92% (vs. 93% in NIO-MET and 74% in MET). These findings clearly demonstrate that co-encapsulation in niosomes significantly enhances sustained and controlled release compared to free or single-loaded formulations.

 To mathematically substantiate the drug transport mechanisms within the niosomes, the in vitro dissolution data were evaluated using the Higuchi matrix diffusion model (Q_t_ = K_H_ *t ^0.5^). When evaluating the dual-loaded formulation (NIO-CUR-MET), the full 72-hour curve yielded a non-linear profile due to the pronounced initial burst release. However, isolating the sustained release phase (8 to 72 h) revealed an exceptional mathematical fit to Higuchi kinetics, with high linearity coefficients for both encapsulated therapeutics (R^2^ = 0.968) for CUR and (R^2^ = 0.985) for MET). This highly linear relationship confirms that after the initial surface-adsorbed drug fraction is depleted, the core release of both lipophilic CUR and hydrophilic MET is strictly governed by a controlled matrix diffusion mechanism through the non-ionic surfactant bilayer^2^.

###  Fourier-Transform Infrared Spectroscopy (FTIR)

 The FTIR spectra of pure drugs, physical mixtures, and formulations are presented in (Figure 5A & 5B). MET: N–H stretching (3379 cm⁻¹), C = N stretching (1622 cm⁻¹), and C–N stretching (1182 cm⁻¹). CUR: O–H stretching (3431 cm⁻¹), C = O/C = C stretching (1627 cm⁻¹), and aromatic vibrations (1510 cm⁻¹). Physical mixture: Characteristic peaks of both drugs with minimal shifts, confirming no significant interaction. Empty niosomes: O–H stretching (3417 cm⁻¹), C–H stretching (2933 cm⁻¹), and C = O stretching (1625 cm⁻¹). Drug-loaded formulations: Minor peak shifts and broadening, especially in O–H/N–H and C = O/C = N regions, suggesting weak hydrogen bonding or molecular interactions. Dual-loaded vesicles displayed peaks corresponding to both CUR and MET, confirming successful incorporation.

**Figure 5:**
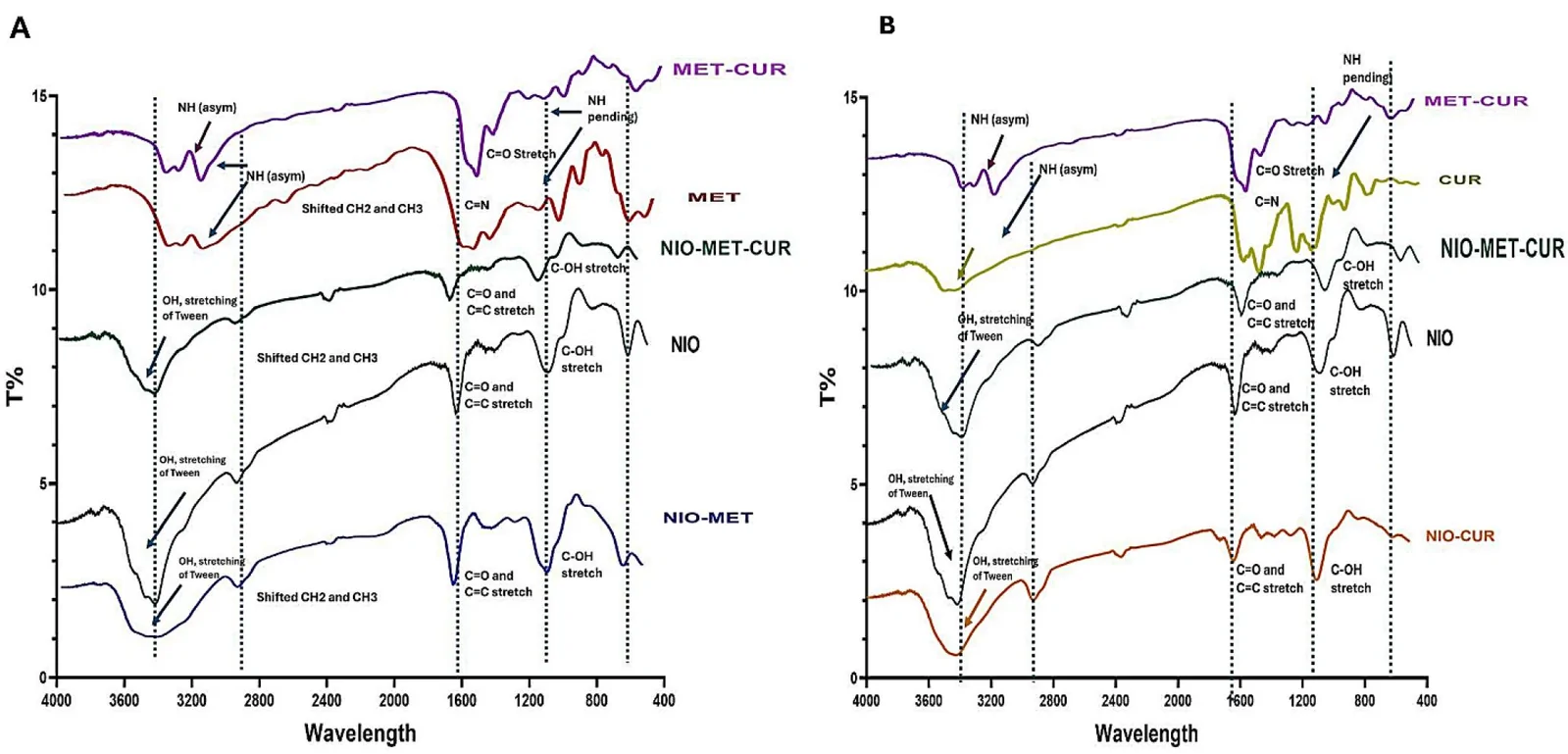
FTIR spectra of (A) MET, MET-CUR, NIO, NIO-MET, and NIO-MET-CUR; (B) CUR, MET-CUR, empty NIO, NIO-CUR, and NIO-MET-CUR

 FTIR analysis provided further confirmation of successful drug encapsulation. Characteristic peaks corresponding to CUR and MET functional groups were detected in the spectra of the loaded formulations, although slight shifts or changes in peak intensity were noted. These changes suggest potential interactions between the drugs and excipients, such as tween 80, or cholesterol, without evidence of chemical degradation, confirming the physical nature of drug loading.^26^

###  Cell Viability (MTT Assay)

 Cytotoxic effects were evaluated in HCT 116 and PANC-1 cells (Figure 6, Table 2). HCT 116 cells: Free CUR had an IC₅₀ of 25 ± 2.2 μM, which decreased to 12 ± 1.5 μM when combined with MET. NIO-CUR further reduced IC₅₀ to 7 ± 0.9 μM, while NIO-CUR-MET showed the strongest effect (4 ± 0.3 μM). MET as a free drug had an IC₅₀ of 3000 ± 100 μM, reduced to 375 ± 56 μM with CUR, 300 ± 41 μM with NIO-MET, and 190 ± 10 μM with NIO-CUR-MET. PANC-1 cells: Free CUR showed an IC₅₀ of 36.5 ± 4.3 μM, improved to 10.1 ± 1.4 μM with MET, 8.4 ± 2.1 μM with NIO-CUR, and 5.2 ± 0.9 μM with NIO-CUR-MET. Free MET exceeded 3000 μM, reduced to 750 ± 56 μM with CUR, 734 ± 41 μM with NIO-MET, and 203 ± 8 μM with NIO-CUR-MET. Encapsulation markedly improved cytotoxicity, particularly for the co-loaded formulation.

**Figure 6:**
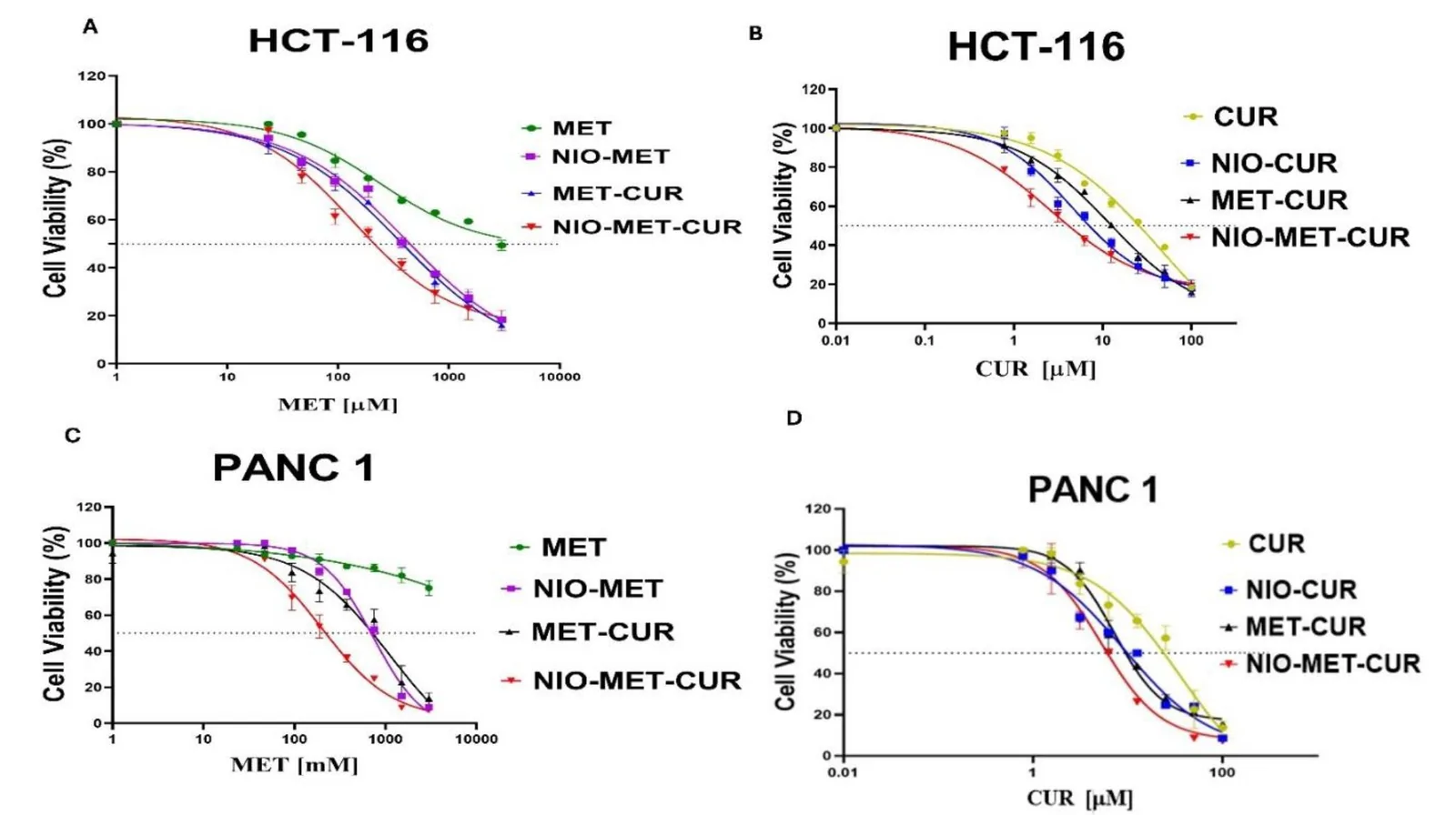
Dose-response curves for cell viability following treatment with free and niosomal formulations. (A) CUR, MET, and CUR-MET in HCT116 cells. (B) NIO-CUR, NIO-MET, and NIO-CUR-MET in HCT116 cells. (C) CUR, MET, and CUR-MET in PANC-1 cells. (D) NIO-CUR, NIO-MET, and NIO-CUR-MET in PANC-1 cells (mean ± SD, n = 3)

**Table 2:** Summary of IC50 values of Free drugs, NIO-MET, NIO-CUR, and NIO-MET-CUR (mean ± SD, n = 3).

	**HCT 116**		**PANC 1**	
**Treatment**	**IC50** **CUR (μM)**	**IC50** **MET (μM)**	**IC50** **CUR (μM)**	**IC50** **MET (μM)**
Single drug	25 ± 2.2	3000 ± 100.2	36.46 ± 4.3	> 3000
CUR-MET	12 ± 1.5	375 ± 56.2	10.1 ± 1.4	750 ± 56.2
NIO (Single drug)	7 ± 9.2	300 ± 40.5	8.4 ± 2.1	734.1 ± 40.5
NIO-CUR-MET	4 ± 3.2	190 ± 10.2	5.2 ± 0.9	202.5 ± 8.2

*In vitro* cytotoxicity studies on HCT116 and PANC-1 cell lines revealed that the NIO-CUR-MET exerted a significantly greater inhibitory effect on cell viability compared to individual drug-loaded or free drug formulations. This enhanced cytotoxicity is likely due to enhanced effect between CUR and MET, both known to interfere with different cellular pathways involved in cancer progression, ^18,19^. CUR induces apoptosis through mitochondrial pathways and ROS generation, while MET activates AMPK, resulting in inhibition of mTOR signalling, contributing to anti-proliferative effects.

###  Apoptosis (Caspase 3/7 Activity)

 Caspase 3/7 activity was assessed after 48 h treatment (Figure 7A–B). HCT 116 cells: Free CUR modestly increased caspase activity, whereas free MET showed minimal effect. The free combination elevated activity ~2.7-fold. NIO-CUR and NIO-MET further enhanced activation, while NIO-CUR-MET achieved the strongest effect (6.5-fold increase). PANC-1 cells: Free drugs showed limited effect, but the combination increased caspase activity ~2.6-fold. NIO-CUR and NIO-MET induced fold increases of 2.8 and 1.6, respectively. Again, the dual-loaded formulation produced the most pronounced response (5.3-fold, *P* < 0.001). These findings confirm that dual-loaded niosomes significantly amplify apoptosis induction.

**Figure 7:**
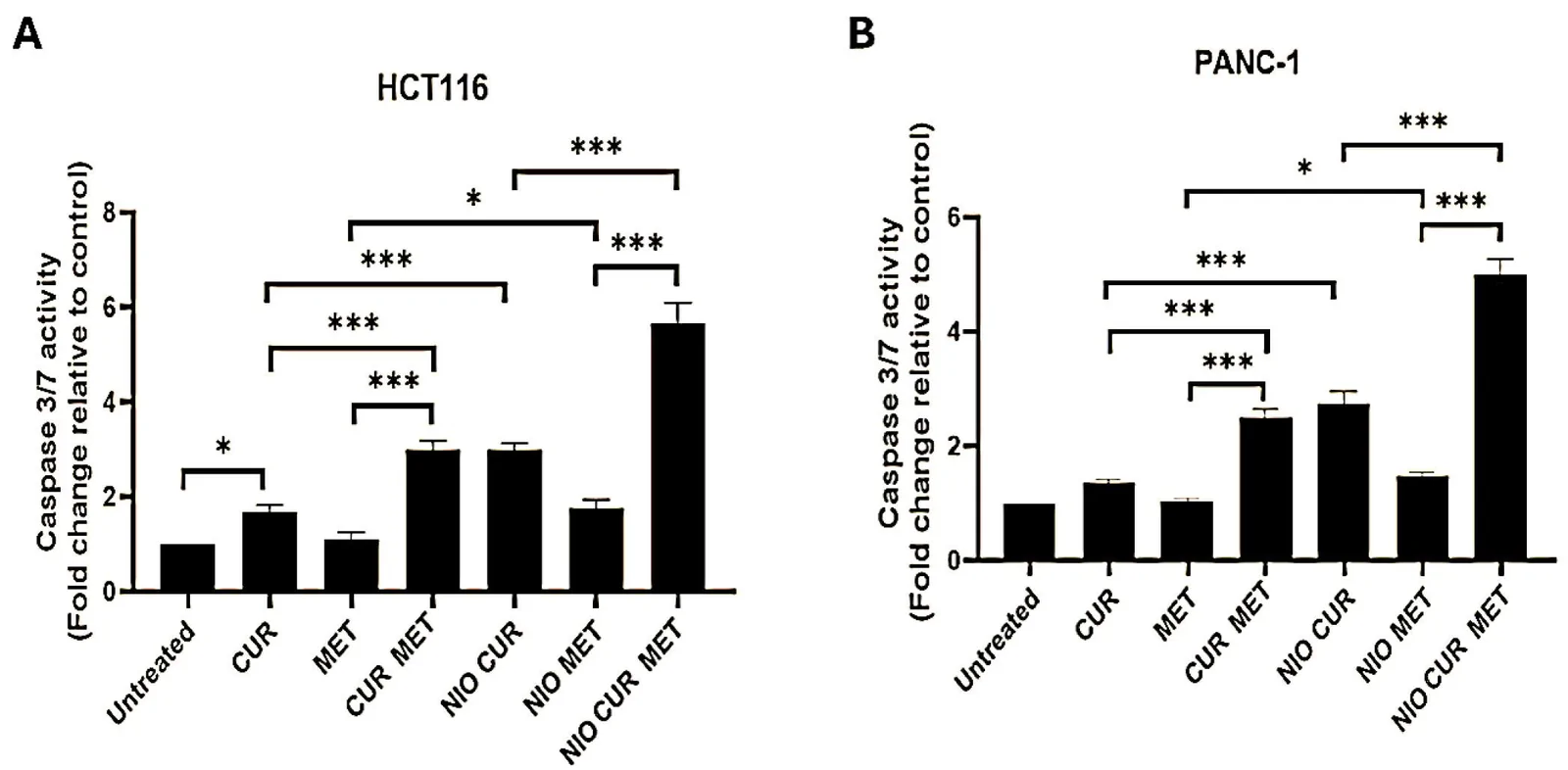
Caspase 3/7 activity in (A) HCT116 and (B) PANC-1 cells after treatment with free and niosomal formulations of CUR and MET (one-way ANOVA followed by Tukey’s post-hoc test, mean ± SD, n = 3, *P*-value < 0.05)

 While Caspase-3/7 activity represents a single endpoint, it is universally recognized as the essential executioner phase of both the intrinsic and extrinsic apoptotic pathways. Its activation directly leads to the structural dismantling of the cell. Therefore, a significant upregulation of Caspase-3/7 activity is a definitive, enzymatically specific indicator of apoptosis rather than non-specific necrosis

###  Anti-Inflammatory Activity

 Levels of TNF-α and IL-1β were quantified in HCT 116 and PANC-1 cells after 24 h treatment (Figure 8A–D). HCT 116 cells: Free CUR reduced TNF-α and IL-1β, while free MET showed minimal effect. The free combination further lowered cytokine levels. Niosomal formulations significantly enhanced suppression, with NIO-CUR-MET producing the strongest inhibition. PANC-1 cells: Similar results were observed, with free CUR effective, MET weak, and combinations superior. Encapsulation further amplified cytokine suppression, and NIO-CUR-MET demonstrated the greatest anti-inflammatory activity, reducing TNF-α to < 50 pg/mL and markedly lowering IL-1β.

**Figure 8:**
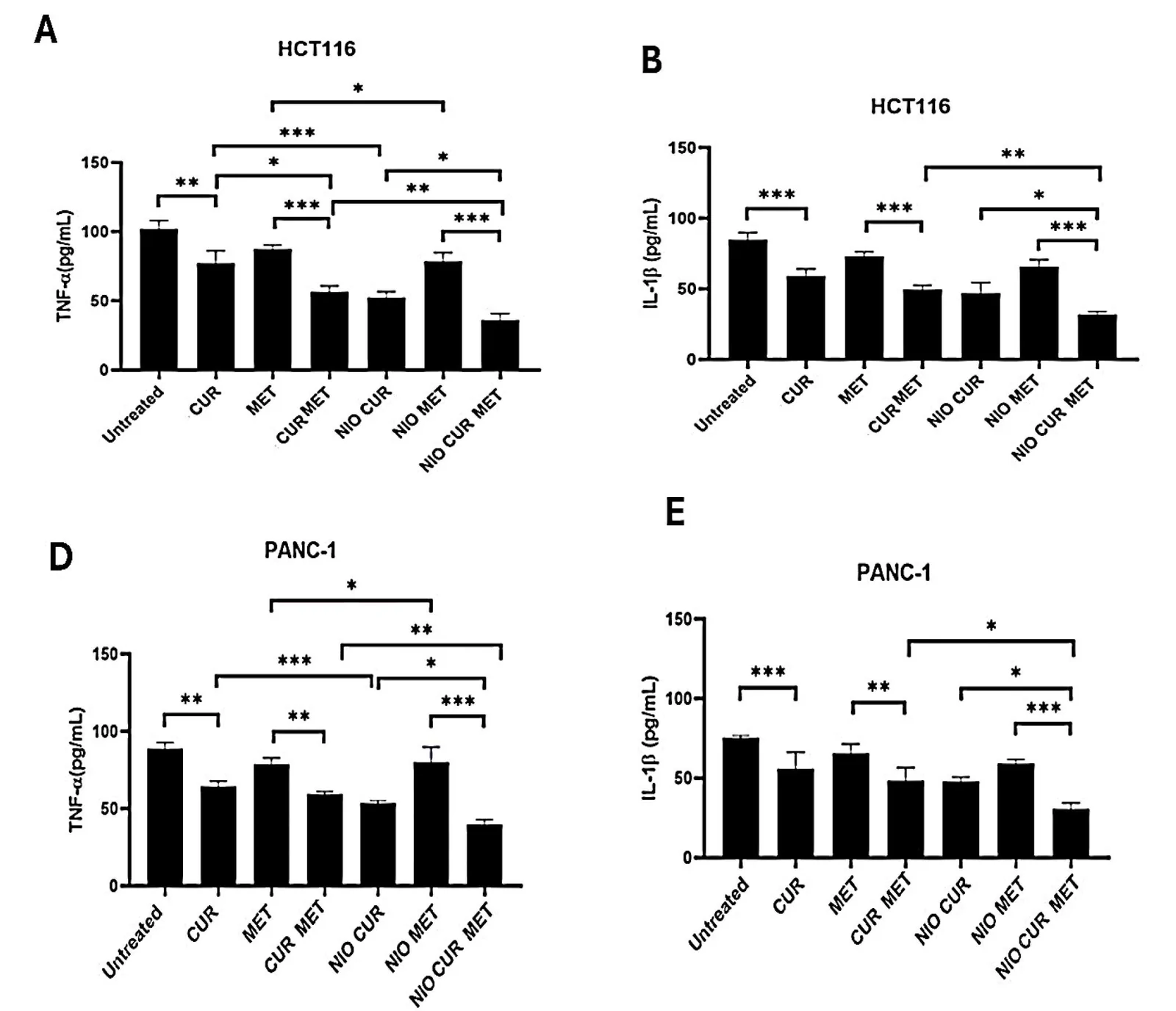
Effect of free and niosomal formulations of CUR and MET on TNF-α and IL-1β production. (A, B) HCT116 cells; (C, D) PANC-1 cells (one-way ANOVA followed by Tukey's post-hoc test, mean ± SD, n = 3, *P*-value < 0.05)

 Apoptosis was evidenced by increased caspase-3/7 activity in cells treated with NIO-CUR-MET, with a more pronounced effect observed in HCT116 cells. These results suggest that the formulation not only enhanced drug delivery but also promoted intracellular pathways associated with programmed cell death. The niosomal system likely improved cellular uptake and prolonged intracellular retention of the drugs, thereby amplifying apoptotic signalling.^27,28^ The combination therapy led to a reduction in TNF-α levels, indicating a potential anti-inflammatory effect, which is particularly important given the role of chronic inflammation in cancer progression. Both CUR and MET are known to possess anti-inflammatory properties, and their co-delivery may enhance therapeutic outcomes by modulating the tumor microenvironment in addition to exerting direct cytotoxic effects.^29-33^

 The present study is subject to several limitations, specifically, there are no in vivo assays which do not provide sufficient information about a tumor. In addition, it is necessary to conduct further analyses concerning advanced characterization and stability of the proposed drug carrier. Moreover, the variation in the encapsulation of drugs can influence the effectiveness of the nanocarrier, since there is considerable fluctuation in the solubility of drugs. Furthermore, the exact mechanism of the synergistic effect has not been identified.

## Conclusion

 Niosomes loaded with CURand MET were successfully prepared using a straightforward ethanol injection method. The resulting formulation exhibited highly satisfactory physical properties, including a small particle size, a stable surface charge, and a highly uniform size distribution. Successful entrapment of both therapeutics within the carriers, without any deleterious alterations to their chemical structures, was confirmed by FTIR analysis. Although the two drugs displayed distinct behavior inside the niosomal matrix, excellent loading levels were achieved for MET alongside highly satisfactory entrapment for the less soluble curcumin.

 The dissolution behavior of both compounds was significantly altered by the niosomes, resulting in a biphasic release profile that extended up to 72 hours. Furthermore, the simultaneous incorporation of CURand MET within a single vehicle led to a cooperative effect, which produced an enhanced anti-proliferative response against HCT-116 and PANC-1 cancer cells compared to the free, unencapsulated drugs.

 Overall, this preliminary investigation demonstrates that niosomal nanocarriers offer a practical approach for the co-delivery of water-soluble and water-insoluble compounds. While the *in vitro* data indicate improved cell-killing efficiency and stable formulation characteristics, this work represents an early-stage screening step. Future studies utilizing flow cytometry will be essential to map out upstream apoptotic markers, such as mitochondrial membrane potential shifts and to investigate downstream cellular signalling. Furthermore, extensive hemocompatibility tests, serum stability profiles, and *in vivo* animal experiments remain strictly necessary to evaluate the true safety, behavior, and potential future application of this co-delivery system.

## Competing Interests

 The authors declare that they have no competing interests.

## Ethical Approval

 This study did not involve human participants or animal subjects. Therefore, ethical approval was not required. All experimental procedures were conducted in accordance with standard laboratory guidelines and regulations.
